# INSIDER: alignment-free detection of foreign DNA sequences

**DOI:** 10.1016/j.csbj.2021.06.045

**Published:** 2021-06-29

**Authors:** Aidan P. Tay, Brendan Hosking, Cameron Hosking, Denis C. Bauer, Laurence O.W. Wilson

**Affiliations:** a Australian e-Health Research Centre, Commonwealth Scientific and Industrial Research Organisation, New South Wales, Sydney, Australia; b Department of Biomedical Sciences, Macquarie University, New South Wales, Sydney, Australia; c Applied BioSciences, Faculty of Science and Engineering, Macquarie University, New South Wales, Sydney, Australia

**Keywords:** Integrated DNA, k-mers, Alignment free, Gene drive, Genomic signature, Anti-microbial resistance detection, Viral integration

## Abstract

External DNA sequences can be inserted into an organism’s genome either through natural processes such as gene transfer, or through targeted genome engineering strategies. Being able to robustly identify such foreign DNA is a crucial capability for health and biosecurity applications, such as anti-microbial resistance (AMR) detection or monitoring gene drives. This capability does not exist for poorly characterised host genomes or with limited information about the integrated sequence. To address this, we developed the INserted Sequence Information DEtectoR (INSIDER). INSIDER analyses whole genome sequencing data and identifies segments of potentially foreign origin by their significant shift in k-mer signatures. We demonstrate the power of INSIDER to separate integrated DNA sequences from normal genomic sequences on a synthetic dataset simulating the insertion of a CRISPR-Cas gene drive into wild-type yeast. As a proof-of-concept, we use INSIDER to detect the exact AMR plasmid in whole genome sequencing data from a *Citrobacter freundii* patient isolate. INSIDER streamlines the process of identifying integrated DNA in poorly characterised wild species or when the insert is of unknown origin, thus enhancing the monitoring of emerging biosecurity threats.

## Graphical abstract

**Figure:**
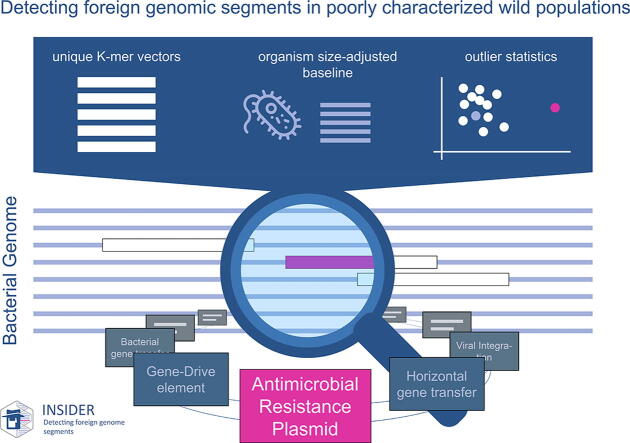

## 1 Introduction

An organism can acquire new external DNA sequences that may insert themselves into the host’s genome. This can occur though natural means such as retroviral insertion, horizontal gene transfer amongst bacterial populations, or through targeted strategies such as the insertion of synthetic DNA sequences using gene-editing technologies [1], [2], [3]. Expression of these foreign DNA sequences can introduce new traits or alter existing traits of the recipient organism. Being able to monitor the acquired sequences by distinguishing foreign from host genome is vital for a range of health, ecological, and environmental applications, such as monitoring the spread of anti-microbial resistance (AMR) or monitoring genetic changes in wild populations [4].

Identifying inserted segments is trivial if the identity of the foreign DNA sequence is known, or if the host genome is well characterised [5]. However, this information is often unavailable in many biosecurity contexts. Instead, entire genomes’ worth of read data must be analysed to identify potentially foreign DNA. This is time-consuming and complicated, requiring significant manual processing. New methods are therefore needed for streamlining the process and enabling more targeted analysis.

In metagenomics, genomic signatures are used to separate out sequences that originate from different species [6]. A genomic signature is comprised of the different k-mer frequencies observed across a genome. Phylogenetically related species are known to have similar signatures, while more distantly related species have more distinct signatures [7], [8], [9]. Inserted sequences from an evolutionary distant or synthetic origin will have signatures that are sufficiently different and therefore will be detectable. This approach significantly reduces the search space from an entire genome to a more focused selection of potential sequences of interest.

Several tools for identifying integrated sequences in the genome have been developed. Notably, Dufraigne *et al.*, [22] Tsirigios *et al*. [23] and Cong *et al*. [24], [25] all describe alignment-free approaches for identifying integrated sequences in the genome. However, these methods rely on prior knowledge about the genome or the inserted sequence. For example, to identify foreign genes or clusters of gene sequences, Tsirigios *et al.* required gene annotation information such as the start and stop positions of genes. Meanwhile, Cong *et al*. required knowledge of the genomes and the phylogenetic relationships among these genomes to identify foreign sequence segments. Furthermore, the performance of these methods on sequencing data of poorly annotated or newly sequenced host genomes has not been reported.

Overcoming these limitations, we developed the INserted Sequence Information DEtectoR (INSIDER) for identifying foreign sequences in the genome. INSIDER converts variable-length sequences into fixed-length frequency vectors (genomic signatures). By analysing the sequence signatures of segments and comparing them to the global observed k-mer signature of that organism, sequence clusters from a different origin can be identified. To illustrate how INSIDER can be used to identify foreign sequences in the genome, we present case studies from yeast and bacteria.

## 2 Materials & methods

### 2.1 Simulated genomic data

We constructed a synthetic data set to simulate the insertion of an RNA-guided gene drive into the genome. To do this, we combined an experimental sequencing library for yeast (Giordano *et al.* [10]) with synthetic reads from the SpCas9 gene. Giordano *et al.* sequenced the genome of wild-type *Saccharomyces cerevisiae* (yeast) strain S288C to a coverage of 80X coverage, resulting in 3.1 million 150 bp paired-end Illumina MiSeq reads (ENA run accession number ERR1938683). Synthetic paired-end reads were generated from the SpCas9 gene (NCBI accession number AAK33936) using ART [11] with software parameters that were consistent with the setup used by Giordano *et al.*

### 2.2 Experimental genomic data

An experimental sequencing library for bacteria was obtained from Peter *et al.* [12] containing hospital patient specimens of *Citrobacter freundii*, an emerging carbapenem‐resistant *Enterobacteriaceae* in Europe (ENA run accession number ERR3307228). They sequenced the genome of an isolate on an Illumina MiSeq sequencing platform. Through *de novo* genome assembly, Peter *et al.* demonstrated that the isolate of *C. freundii* had acquired the plasmid harbouring antibiotic resistant genes from *Pseudomonas aeruginosa*.

### 2.3 Identification of foreign DNA sequences using INSIDER

INSIDER (v1.00) was developed in Python and is used as a command-line tool. The source code is available via the Github: https://github.com/aehrc/INSIDER under the GPL v3 license.

**Fig. 1:**
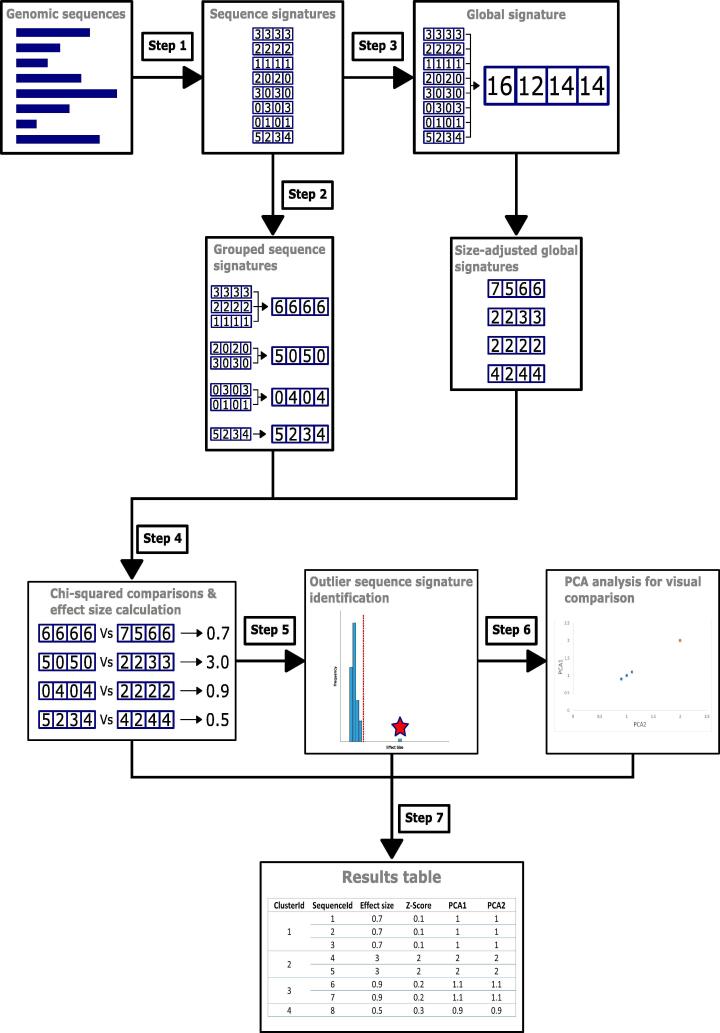
Overview of INSIDER. Full description in methods. 1) and 2) Unique k-mer frequencies are calculated. 3) Size-adjusted global average signatures are calculated. 4) Effect sizes are generated. 5) Foreign sequences are identified as outliers. 6) Evolutionary distances of signatures are visualised as PCA plot. 7) Results are tabularized for machine readable information access.

An overview of INSIDER is shown in Fig. 1. The tool requires a list of genomic sequences in FASTA format. Sequences can be fully sequenced genomes, contigs from genome assembly or sequenced reads. Analysing the input sequences with INSIDER involves the following steps. 1) Variable length sequences in the FASTA file are converted into fixed-length frequency vectors (referred to as sequence signatures). This is done by identifying all possible subsequences of a given length (i.e., k-mer), and counting the frequency of each k-mer. 2) We merge all identical frequency vectors to create unique signatures, which are then clustered by first performing dimensionality reduction with t-SNE [13] and then grouping with DBSCAN [14]. 3) A global signature for the organism is created by generating the k-mer frequency over the whole genome. We then create size-adjusted global averages to match the observed contig lengths. 4) The signature of each unique cluster is then compared to the respective size-adjusted global average to determine the effect size using Chi-square ($\chi^{2}$) goodness-of-fit tests. This approach was chosen over p-value calculation because relatively small differences across a relatively high-dimensional frequency vector can lead to inflated p-values. Sequence signatures associated with foreign DNA sequences will have a large effect sizes, while sequence signatures associated with the host genome are likely to have relatively small effect sizes. 5) To detect the foreign sequence clusters, we identify outliers based on the observed distribution of effect sizes, where Z-score > 1 were considered outliers. 6) To visualise the genomic distance between each contig, we perform PCA on the signatures of each unique cluster and the global signature, and plot the first two components of PCA. PCA was used instead of t-SNE since pairwise distances in t-SNE may not reflect the dissimilarity between unique sequence signatures. 7). The results of step 4, 5 and 6 are recorded in the output tab-separated (TSV) file.

## 3 Results

### 3.1 Separation of genomes using k-mer signatures

We first confirmed that k-mer signatures can distinguish between sequences originating from genomes of distinct organisms. One hundred 10 kb sequences were randomly generated from each of the yeast (*Saccharomyces cerevisiae*), fruit fly (*Drosophila melanogaster*), zebrafish (*Danio rerio*), mouse (*Mus musculus*) and human (*Homo sapiens*) genomes. All five hundred subsequences were then converted into sequence signatures using 5-mers, and subsequently clustered using t-SNE. Clusters were then defined using DBSCAN.

**Fig. 2:**
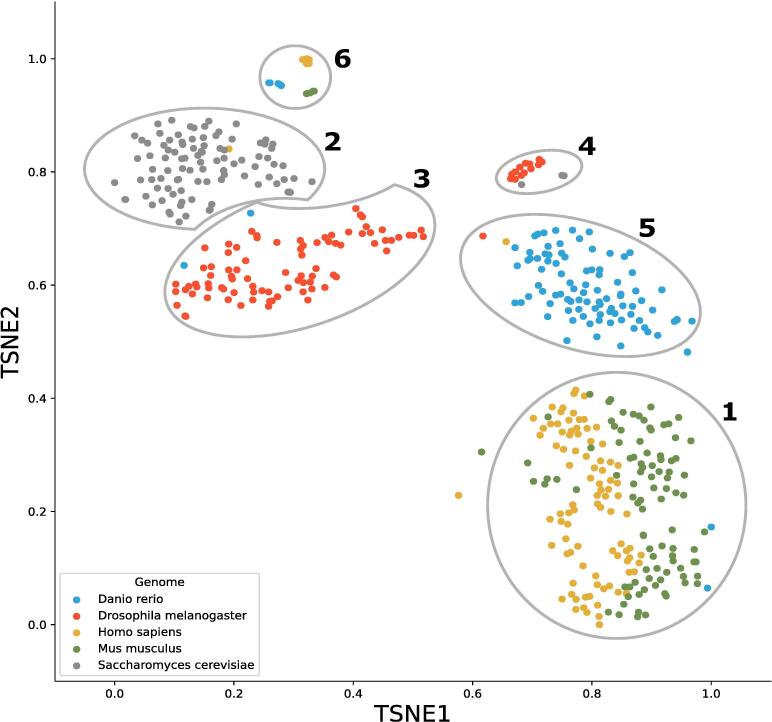
t-SNE plot showing sequences from different eukaryotic genomes. Each point represents a 10 kb subsequence from a eukaryotic genome, and points are highlighted based on their eukaryotic genome. For groups containing fewer than 10 sequences, no label or group assignment is shown.

Visualization of the results revealed several distinct groups, with between 1 and 186 sequences in each group (Fig. 2). We investigated the composition of groups with>10 sequences and found most to be homogeneous. Specifically, groups 2, 3 and 5 contained yeast (96/97 or 99%), fruit fly (85/86 or 99%) and zebrafish (88/89 or 99%) genomes, respectively. In contrast, group 1 was heterogeneous containing sequences from both mouse (95/189 or 50%) and human (89/189 or 48%), marking the detection limit for k-mer approaches to differentiate between closely related species. A special case of this relatedness is cluster 4 and 6, which contained the mitochondrial DNA of each eukaryotic genome. Mitochondrial DNA has a low compositional similarity to nuclear DNA, but a high similarity between mitochondrial DNA of different species [15].

To test the effects of shorter sequences and k-mer lengths, we repeated the analysis using one hundred subsequences of different lengths (ranging between 0.1 kb and 10 kb) from each genome and different values of K (ranging between 1 and 8). We found that the shortest sequence to result in unique clusters was 2 kb (Supplementary Fig. 2A), below that k-mer signatures are too unstable to result in distinct clusters as exemplified in Supplementary Fig. 1 for sequences of 1 kb. This implies that including shorter sequences will lead to poorer performance and that slightly longer subsequences (i.e., > 1 kb) will be sufficient for establishing distinct clusters in most cases. Similarly, we identified a K of 5 led to the most homogeneous clusters, offering a good trade-off between sequence specificity and computational resources (Supplementary Fig. 2B).

Together, our results demonstrate that sequences from different eukaryotic genomes can be distinguished based on their signatures and highlight the capacity of INSIDER for identifying foreign DNA sequences.

## 4 Case study 1: Detecting the presence of synthetic gene drive sequences

Having established that k-mer signatures can separate distinct genomes, we then investigated whether INSIDER could identify an integrated sequence. In this case study, we investigated whether INSIDER could identify gene drive elements, inserted into a yeast genome. Gene drives are “selfish” genetic elements that have a higher chance of being inherited than normal alleles and hence spreading through a population within several generations [4]. Research is conducted into their application to combat biosecurity threats (e.g. prevent the spread of diseases by controlling mosquito populations) [16], [17], [18]. The most common form of artificial gene drives leverages the CRISPR-Cas system for gene editing [4]. Due to its bacterial origin, the CRISPR-Cas gene likely has a unique genomic signature. We constructed a synthetic dataset to simulate the insertion of a CRISPR-Cas gene (the main component of a CRISPR-Cas gene drive [4]) into the genome. The dataset was constructed by combining an experimental sequencing library for wild-type yeast strain S288C (Giordano *et al.* [10]) with synthetic reads from the SpCas9 gene (simulated using ART [11], see methods for more detail). Because short sequences were shown to confound the results in the prior section, paired-end reads were *de novo* assembled into contigs with SPAdes [19] using default parameters, and then all contigs smaller than 2 kb were subsequently removed, resulting in 145 contigs, with a size range of 2,081 to 518,057 bases.

We calculated the k-mer signature for each contig and clustered similar signatures. For each cluster, we calculated the average signature. Cluster averages were then compared to their respective size-adjusted global average using Chi-square ($\chi^{2}$) goodness-of-fit tests, with more divergent clusters giving a high effect size.

**Fig. 3:**
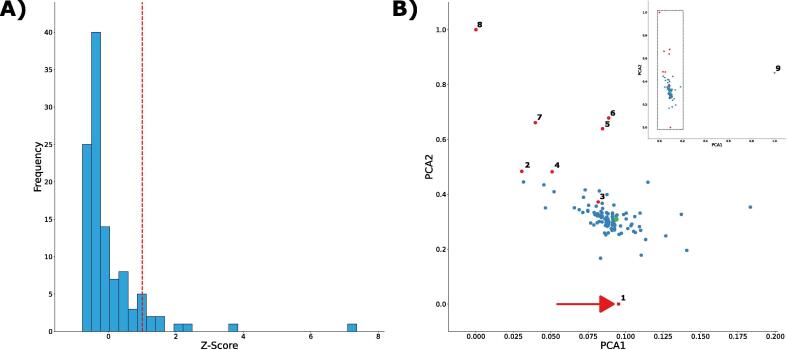
A) Histogram of contig signature Z-scores. Contig signatures with Z-scores > 1 were considered outliers. B) PCA plot of contig signatures. Each point represents a unique contig signature. The estimated genome signature is highlighted in green and outlier contig signatures are shown in red. The contig signature of the SpCas9 gene sequence (indicated with the red arrow) was considered an outlier. Each cluster is numbered according to Table 1. (For interpretation of the references to colour in this figure legend, the reader is referred to the web version of this article.)

Rather than thresholding using an effect size cut-off, we elected to calculate the Z-score for each cluster, measuring how different the associated effect size was from the average effect size over all contigs (Fig. 3A). We define clusters with Z-score > 1 as outliers.

The divergence amongst clusters was then visualised using the first two components of the Principle Component Analysis (PCA) which, unlike t-SNE, conserves the distance between points and is therefore more suited for visualizing outliers (Fig. 3B). In total, 9 clusters were identified as being significantly different from the global average (Table 1). These included both the expected mitochondrial cluster, as well as the CRISPR-Cas9 contig, confirming that INSIDER can identify foreign DNA sequences.

**Table 1:** Identity of significant clusters.

Contig Signature ID	Z-Score	Description
1	1.463267	SpCas9 Gene
2	1.129249	Protein of unknown function
3	1.203519	2-µm plasmid
4	1.006398	Transposable element (Retrotransposon; similar to retroviral genes)
5	1.604037	Pseudogene
6	2.179955	Protein of unknown function
7	2.27638	Cell surface glycoprotein
8	3.680128	Protein of unknown function
9	7.362642	Mitochondrial genome

We aligned the sequences of the remaining clusters to the yeast genome to identify their identity. One contig was identified as originating from the 2-µm plasmid, a known selfish genetic element which spreads through yeast [20], and another contig was identified as a transposable element likely originating from a retrotransposon or retrovirus. The remaining five contigs mapped to various genes or pseudogenes endogenous to yeast.

Together, the above results demonstrate that INSIDER can streamline the process of identifying integrated DNA, reducing the search space from the entire genome to only targeted sequences, and requires no prior knowledge about the genome or inserted sequence. This offers the ability to monitor gene drive spread in the wild.

### 4.1 Case study 2: Detecting the presence of antibiotic resistance sequences

We next investigated whether INSIDER could identify an acquired plasmid amongst bacterial sequences, using sequencing data from a *C. freundii* patient isolate known to contain an anti-microbial resistance (AMR) encoding plasmid [12]. In the original paper, the plasmid was detected through reassembly of the bacterial and plasmid genomes using the *pathoLogic* pipeline and alignment to known AMR sequences [12]. For this study however, we assumed no prior knowledge about the genome of the bacteria or the sequence of the plasmid and investigated whether INSIDER could detect the presence of a foreign DNA element. We analysed the data using the same protocol as previously, using the same Z-score threshold of 1.

**Fig. 4:**
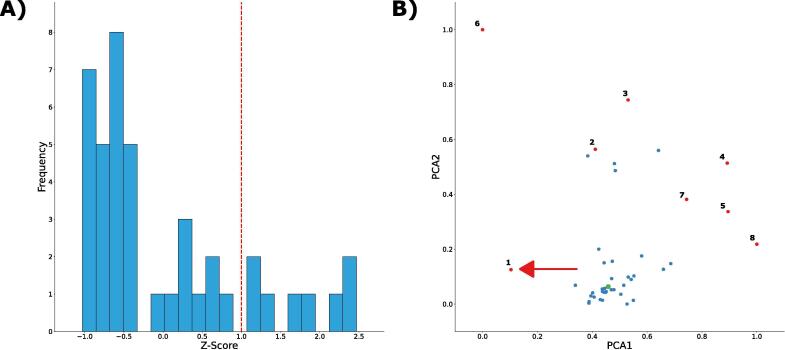
A) Histogram of contig signature Z-scores. Contig signatures with Z-scores > 1 were considered outliers. B) PCA plot of contig signatures. Each point represents a unique contig signature. The estimated genome signature is highlighted in green and outlier contig signatures are shown in red. The contig signature of the plasmid sequence (indicated with the red arrow) was considered an outlier. Each cluster is numbered according to Table 2. (For interpretation of the references to colour in this figure legend, the reader is referred to the web version of this article.)

INSIDER analysed 46 assembled contigs (>2kb), with a size range of 2,437 to 444,812 bases. Unlike the previous eukaryote sequences, the majority of bacterial contigs were sufficiently unique to be classified as induvial clusters, with the 46 contigs resolving into 42 clusters suggesting that there was little repetitiveness within the bacteria’s genome. Through comparison to the global average, 8 of these clusters were flagged as significantly different (Fig. 4). Having prioritized the candidates of interest, we were then able to determine the identity of these clusters by aligning them to the non-redundant nucleotide database using NCBI BLAST [21]. Using this species and domain-agnostic approach, we were able to identify, the same AMR-encoding plasmid as identified by the original paper, the SDENCHOLpb plasmid (Table 2). For the remaining 7 significant outliers, the BLAST search annotated them to be from other species of bacteria, or from other strains of *C. freundii* suggesting that horizontal gene transfer had occurred in this population multiple times in the past.

**Table 2:** Identity of significant clusters.

Contig Signature ID	Z-Score	Top hit	Originating Organism
1	2.388083	Sterolibacterium denitrificans strain Chol genome assembly, plasmid: SDENCHOLpb	*Sterolibacterium denitrificans*
2	1.195658	Escherichia coli strain ECONIH5 chromosome, complete genome	*Escherichia coli*
	1.195658	Citrobacter freundii strain RHBSTW-00135 chromosome, complete genome	*Citrobacter freundii*
3	1.284483	Citrobacter freundii strain RHB30-C03 chromosome, complete genome	*Citrobacter freundii*
4	1.708026	Enterobacter cloacae STN0717-60 DNA, complete genome	*Enterobacter cloacae*
5	1.777462	Citrobacter freundii strain RHBSTW-00370 plasmid pRHBSTW-00370_4, complete sequence	*Citrobacter freundii*
6	2.211883	Escherichia coli strain MS6192 chromosome, complete genome	*Escherichia coli*
7	1.15233	Escherichia coli strain ECONIH4 chromosome, complete genome	*Escherichia coli*
8	2.469612	Anderseniella sp. Alg231_50 genome assembly, chromosome: VII	*Anderseniella* sp. *Alg231-50*

## 5 Discussion

In this study we presented INSIDER, a tool for identifying integrated sequences within genomes. INSIDER works by converting variable-length sequences into fixed-length k-mer frequency vectors (referred to as genomic signatures). By analysing these sequence signatures, sequences that are likely to have originated from a different genome (i.e., foreign sequences) can be identified. INSIDER is the first tool specifically designed to specifically function with no prior knowledge about the genome, meaning it can be readily used to analysed completely novel genomes.

As a proof of concept we demonstrated how INSIDER can be used to identify the presence of both a CRISPR-Cas9 gene within a genome (a hallmark of an artificial gene drive) as well as detect the presence of an AMR-encoding plasmid in a bacterial sample. In both cases, INSIDER required no prior knowledge about the sequences of either the host genome or integrated DNA, making it an ideal monitoring tool in the biosecurity context, where genomes are often poorly annotated and the identity of an integrated sequence is unknown. In addition to identifying the foreign material, INSIDER also flagged several endogenous sequences, such as the mitochondrial genome, as significantly different to the global average. In the future, incorporating some of the more common unique genomic signatures may allow for better separation between significant and expected genomic signature variation. Regardless, our pipeline was able to successfully reduce the search space from the entire genome to only a handful of targeted sequences allowing for more targeted and streamlined downstream analysis.

While INSIDER was able to separate out sequences from distinct genomes, sequences from more related species (e.g., human and mouse) showed significant similarities in their global signatures, making their separation difficult. Refinement of the k-mer pipeline, e.g. through varying k-mer length, may improve the resolution and specificity of the INSIDER pipeline [26]. Using longer k-mers, it is possible to separate out sequences from more evolutionary similar species [27]. Longer k-mers may also provide a means to properly characterise shorter sequences, which are more susceptible to smaller changes in k-mer frequency [28]. However, this increase in specificity must be balanced with a significant increase in the associated computational costs, as increasing the k-mer length increases the number of associated features that must be calculated for each sequence. In a biosecurity context however, the integrated sequences of interest often originate from distinct genomes (e.g. retrovirus or artificial construct), meaning the differences can be captured using a shorter k-mer length.

In conclusion, we have developed INSIDER, a tool for identifying foreign sequences in the genome. With INSIDER, we showed that sequences originating from distinct genomes can be distinguished based on their signatures. Through case studies of yeast and bacteria, we demonstrated how INSIDER can be used to identify foreign sequences in the genome. INSIDER is therefore a powerful tool that will streamline the process of identifying integrated DNA of unknown origin in poorly characterised wild species, allowing for enhanced monitoring of emerging biosecurity threats.

## CRediT authorship contribution statement

**Aidan P. Tay:** Conceptualization, Methodology, Software, Formal analysis, Investigation, Data curation, Writing - original draft, Writing - review & editing, Visualization. **Brendan Hosking:** Software, Methodology. **Cameron Hosking:** Software, Methodology. **Denis C. Bauer:** Writing - review & editing, Visualization, Supervision, Project administration. **Laurence O.W. Wilson:** Conceptualization, Methodology, Writing - original draft, Writing - review & editing, Supervision, Project administration, Funding acquisition.

## Declaration of Competing Interest

The authors declare that they have no known competing financial interests or personal relationships that could have appeared to influence the work reported in this paper.
