# Supplementary material for: INSIDER: alignment-free detection of foreign DNA sequences
Source: Comput Struct Biotechnol J. 2021 Jun 29;19:3810–6. doi: 10.1016/j.csbj.2021.06.045 (PMC8273350; doi:10.1016/j.csbj.2021.06.045)
Supplement: Supplementary data 1 [file mmc1.docx]

**SUPPLEMENTARY MATERIALS**


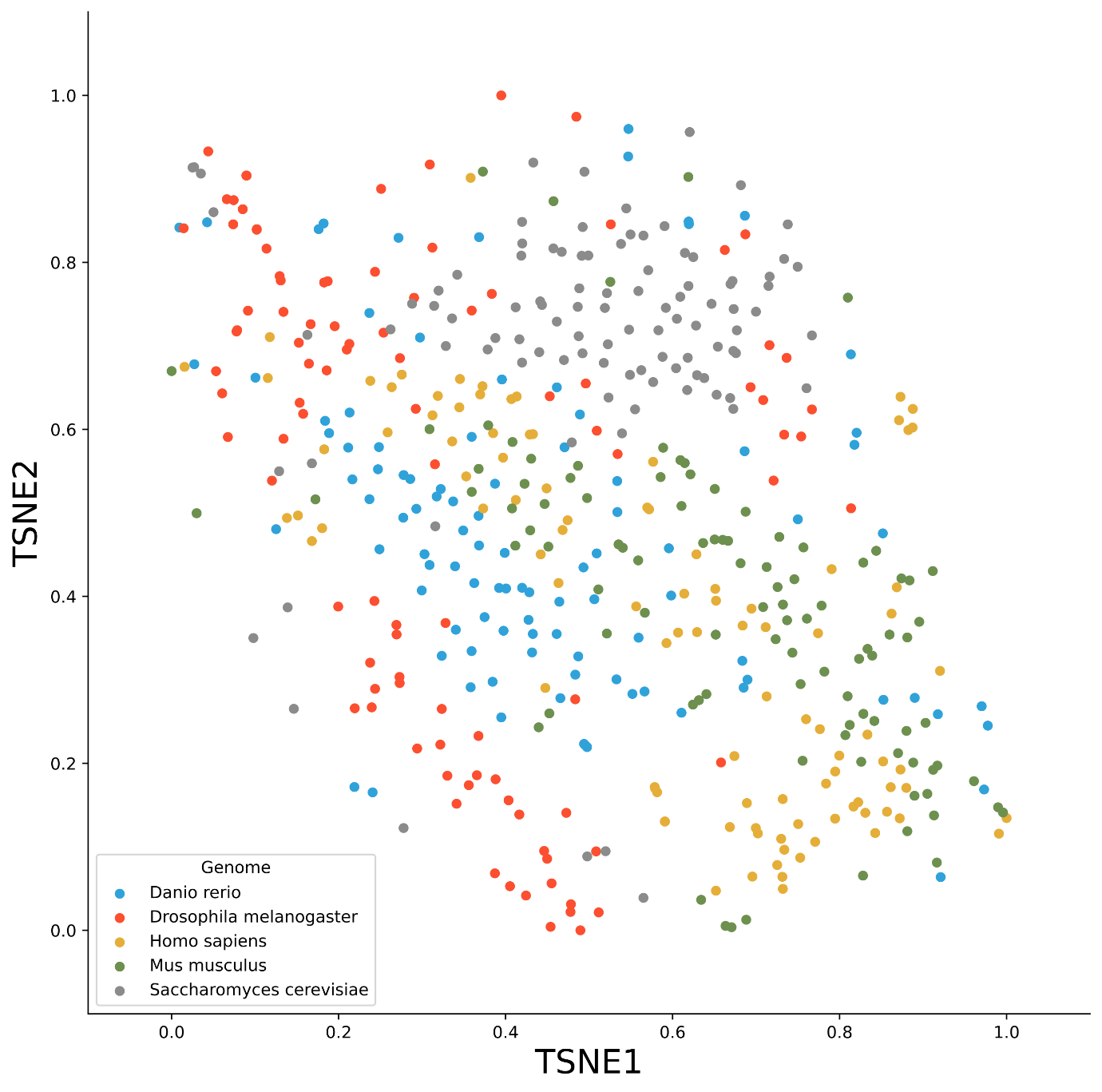


**Supplementary Figure 1:** t-SNE plot showing sequences from different eukaryotic genomes. Each point represents a 1kb subsequence from a eukaryotic genome, and points are highlighted based on their eukaryotic genome. Sequences are scattered throughout the plot, indicating that sequences from the same eukaryotic genome were assigned to different groups.


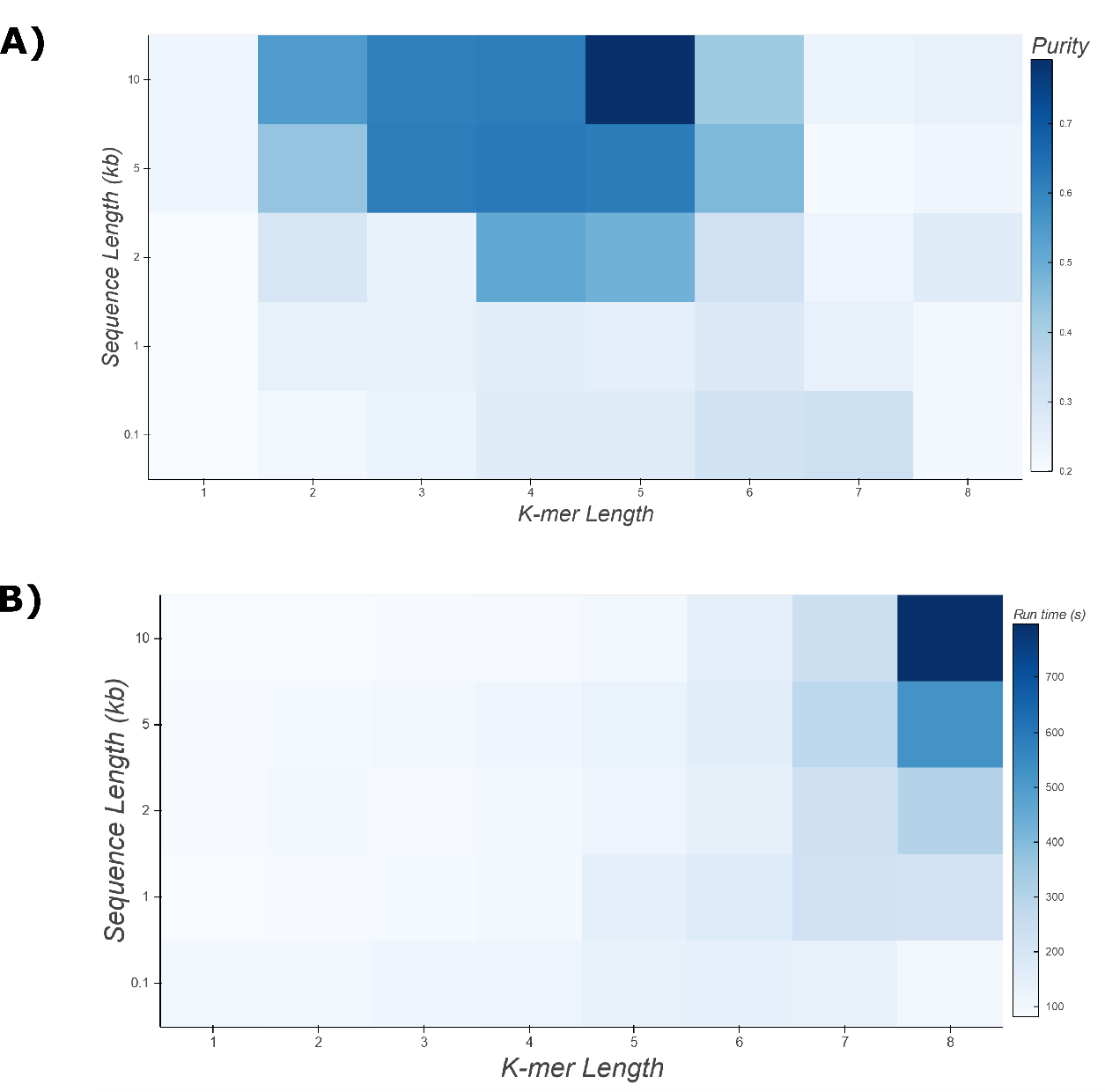


**Supplementary Figure 2:** Heat map showing the results of analyses for different values of K (ranging between 1 and 8) and one hundred subsequences of different length (ranging between 0.1kb and 10kb) from each eukaryotic genome. Each cell in the heat map corresponds to either the A) clustering purity, which measures the homogeneity of groups arising from clustering, or the B) analysis run time. Subsequent clustering of 5-mers led to the highest clustering purity, indicating that the groups were mostly homogeneous and that the unique k-mer profiles for each genome could be established. Clustering of 5-mers also offered a good trade-off between sequence specificity and computational resources.
